# The thresholds for statistical and clinical significance – a five-step procedure for evaluation of intervention effects in randomised clinical trials

**DOI:** 10.1186/1471-2288-14-34

**Published:** 2014-03-04

**Authors:** Janus Christian Jakobsen, Christian Gluud, Per Winkel, Theis Lange, Jørn Wetterslev

**Affiliations:** 1 Copenhagen Trial Unit, Centre for Clinical Intervention Research, Department 7812 Rigshospitalet, Copenhagen University Hospital, Copenhagen, Denmark; 2 Emergency Department, Holbæk Hospital, Holbæk, Denmark; 3 Department of Biostatistics, Faculty of Health Sciences, University of Copenhagen, Copenhagen, Denmark

**Keywords:** Randomised clinical trial, Threshold for significance, Bayes factor, Confidence interval, *P*-value

## Abstract

**Background:**

Thresholds for statistical significance are insufficiently demonstrated by 95% confidence intervals or *P*-values when assessing results from randomised clinical trials. First, a *P*-value only shows the probability of getting a result assuming that the null hypothesis is true and does not reflect the probability of getting a result assuming an alternative hypothesis to the null hypothesis is true. Second, a confidence interval or a *P*-value showing significance may be caused by multiplicity. Third, statistical significance does not necessarily result in clinical significance. Therefore, assessment of intervention effects in randomised clinical trials deserves more rigour in order to become more valid.

**Methods:**

Several methodologies for assessing the statistical and clinical significance of intervention effects in randomised clinical trials were considered. Balancing simplicity and comprehensiveness, a simple five-step procedure was developed.

**Results:**

For a more valid assessment of results from a randomised clinical trial we propose the following five-steps: (1) report the confidence intervals and the exact *P-value*s; (2) report Bayes factor for the primary outcome, being the ratio of the probability that a given trial result is compatible with a ‘null’ effect (corresponding to the *P*-value) divided by the probability that the trial result is compatible with the intervention effect hypothesised in the sample size calculation; (3) adjust the confidence intervals and the statistical significance threshold if the trial is stopped early or if interim analyses have been conducted; (4) adjust the confidence intervals and the *P*-values for multiplicity due to number of outcome comparisons; and (5) assess clinical significance of the trial results.

**Conclusions:**

If the proposed five-step procedure is followed, this may increase the validity of assessments of intervention effects in randomised clinical trials.

## Background

Clinical experience and observational studies cannot and should not be used to validate intervention effects [1]. The randomised clinical superiority trial remains the mainstay of modern clinical intervention research and is needed for a valid assessment of possible causality between interventions and outcomes [1].

Most commonly, the statistical analyses in randomised clinical trials are performed under the frequentist paradigm. In this approach, a significant difference in effect is declared when a value of a test statistic exceeds a specified threshold showing that it is unlikely that the trial results are produced by zero difference in effect between the compared interventions, i.e., that the null hypothesis is true [2]. A *P*-value less than 5% has been the most commonly used threshold for statistical significance in clinical intervention research since Fisher warned against exactly that in 1955 [3-5]. *P*-values are easily calculated but are often misinterpreted [6,7] and misused [8-10].

In the following we describe the methodological limitations of focusing too much on confidence intervals and *P*-values, and suggest a five-step procedure for a more valid assessment of results of intervention effects in randomised clinical superiority trials [11]. Our recommendations do not solve all problems of interpreting results from randomised clinical trials, but we aim to present a valid, practical, relatively simple, and yet comprehensive assessment tool to be used by trial investigators and clinical research consumers. The five following sections of the manuscript will correspond to each step of the proposed five-point assessment.

## Methods and results

### The confidence interval and the *P*-value

Due to stochastic variation (‘play of chance’) in biomedical data, statistical analyses are needed to clarify if the results demonstrate a genuine difference in effect between the compared interventions in a randomised clinical trial [1]. The *P*-value describes the probability of obtaining an observed or larger difference in intervention effect purely by ‘play of chance’ assuming that there is no intervention effect (i.e., assuming that the ‘null hypothesis’ is true) (Additional file 1: Table S1). Trialists can and should report the calculated confidence intervals and the exact *P-*values, but their exclusive relation to the null hypothesis should be kept in mind.

Confidence intervals not containing 1.0 for binary outcomes (or hazard ratios for survival data) or 0.0 for continuous outcomes are, as well as the corresponding *P*-value, often used as thresholds for statistical significance. Reporting confidence intervals have rightfully been claimed a more appropriate and understandable demonstration of the statistical uncertainty [12,13]. However, confidence intervals do not essentially provide more information than implicitly given by the estimated effect and the *P*-value. The confidence interval and the observed effect size can be derived from the *P*-value — and vice versa [14,15]. We believe it is informative both to report the confidence interval and the corresponding exact *P-*value because the former explicitly demonstrates the range of uncertainty of the intervention effect and the latter tells how likely the results are assuming the null hypothesis is true.

### Sample size estimation, the alternative hypothesis to the null hypothesis, and Bayes factor

Before conducting a randomised clinical trial one should estimate the required sample size based on the primary outcome [16-18]. A sample size calculation estimates the number of trial participants necessary to demonstrate or discard a specific *a priori* anticipated intervention effect with specified error probabilities [16]. In order to calculate a sample size relating to one specified primary outcome, it is necessary:

• To define an anticipated difference in intervention effect (i.e., a hypothesis alternative to the null hypothesis) between the compared intervention groups. This intervention effect could, e.g., be a mean difference, an odds ratio, or a hazard ratio [16]. This hypothesised difference in effect should be based on the most realistic intervention effect as suggested by a meta-analysis of prior evidence with low risks of bias (Additional file 1: Table S1) [19,20], but may also be defined as a ‘minimal relevant clinical difference’ (see Statistical significance and clinical significance).

• To estimate the variability of the anticipated difference in intervention effect (e.g., a standard deviation of a mean difference or a proportion of participants with an outcome of interest in the control intervention group).

• To decide on an acceptable risk of falsely rejecting the null hypothesis (alpha or type I error) (most investigators choose 5%, see ‘Discussion’) and an acceptable risk of falsely accepting the null hypothesis (beta or type II error) (most investigators choose 10% or 20%).

The lower the anticipated intervention effect is and the lower the above acceptable risks are, the larger the sample size becomes.

When the estimated sample size has been obtained, the null hypothesis can be tested and rejected if *P* is below 5%. However, a low exact *P*-value may be misleading if there, at the same time, is a low probability of the trial results being compatible with the intervention effect hypothesised in the sample size calculation. To compensate for this deficiency of the *P-*value it is helpful to calculate Bayes factor [21,22], which is the ratio between the probability of getting the result assuming the null hypothesis (H_0_) is true divided by the probability of getting the result assuming the alternative hypothesis (H_A_) is true [21]. In the following we have chosen to quantify the alternative hypothesis (H_A_) (Additional file 1: Table S1) as the intervention effect hypothesised in the sample size calculation, but Bayes factor can (and in some instances should) be defined differently. For an in-depth discussion of Bayesian methods and principles see reference [21].

**Figure 1:**
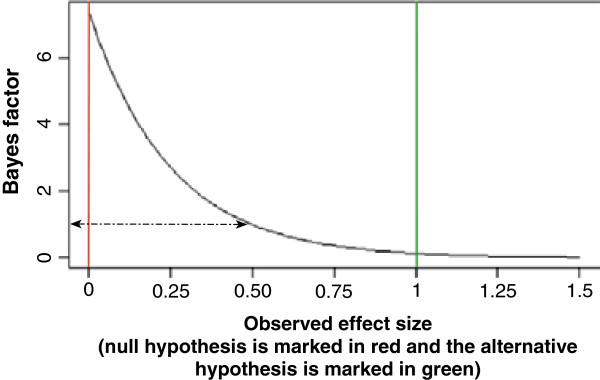
**A figure showing how Bayes factor will change according to different observed effects.** The red left vertical line represents the null hypothesis (an effect of null), the right green vertical line represents an alternative hypothesis to the null hypothesis with an effect of 1.0. The black curve shows that Bayes factor will be 1.0 when the observed effect size if exactly half of the effect size of the alternative hypothesis; and the curve shows that Bayes factor will decease with increasing observed effect sizes.

Figure 1 depicts Bayes factor as a function of the observed effect size, where the observed effect size is expressed as fractions of ‘1.0’. When Bayes factor is 1.0, the likelihoods of the null hypothesis and the alternative hypothesis are the same, i.e., the observed effect size is exactly half way between null effect and the hypothesised effect size. When Bayes factor is less than 1.0, the trial results are more compatible with the alternative hypothesis than the null hypothesis. When Bayes factor is larger than 1.0, the trial results are more compatible with the null hypothesis than the alternative hypothesis.

Confidence intervals not containing 1.0 for binary outcomes (and hazard ratios) or 0.0 for continuous outcomes and low exact *P*-values do not necessarily correspond to a low Bayes factor — and confidence intervals and *P*-values may in some circumstances misleadingly indicate evidence for an intervention effect [19,21]. A low Bayes factor (e.g., less than 0.1) together with a low *P* value (e.g., less than 0.05) will correspond to a high probability of an intervention effect similar to or even greater than the hypothesised intervention effect used in the sample size calculation (Figure 1) (Additional file 1: Table S1).

The intervention effect hypothesised in the sample size calculation should be based on what prior evidence indicates provided prior evidence exists. By calculating Bayes factor as above, new trial results will be related to former evidence. If a new trial result demonstrates an intervention effect closer to zero than prior evidence indicates, a high Bayes factor will demonstrate that there is a low probability that the intervention effect indicated by prior evidence is compatible with the new trial result. On the other hand, the validity of a trial result will increase if a new trial result including a low Bayes factor shows intervention effects similar to (or larger) what prior evidence has indicated, i.e., that the new trial result is compatible with the intervention effect indicated by prior evidence.

If no former trials have been conducted, an anticipated intervention effect cannot be estimated based on empirical high quality data. Anticipated realistic intervention effect may still be chosen based on knowledge about other analogous interventions’ effects on the same disease or condition [23,24], but the uncertainty related to the choice of an anticipated intervention effect prior to the trial conduct and the subsequent estimation of a sufficient sample size remain a problem. The possibility to adjust the hypothesised intervention effect to get low values of Bayes factor makes Bayes factor sensitive to biased *post hoc* analyses. When Bayes factor is to be calculated, it is therefore essential to define the intervention effect hypothesised in the sample size calculation *a priori* so biased *post hoc* analyses can be avoided.

Trialists might be tempted to perform a sample size calculation based on unrealistically large anticipated intervention effects in order to reduce the necessary number of participants in a trial (relatively few patients are needed to demonstrate or discard large intervention effects) [25,26]. However, sample size estimations based on unrealistically large anticipated intervention effects increase the risk of erroneous estimation of intervention effects — as trials with too small sample sizes (relative to the actual effect) have been shown to have an increased risk of either overestimating or underestimating both effect size and variability [27]. This also means that the calculation of Bayes factor before a realistic sample size has been reached will also be relatively unreliable, because the observed effect size used to calculate Bayes factor might be erroneous (Additional file 1: Table S1). Bayes factor assessed after the sample size has been reached will increase if the trial results show an intervention effect smaller than the intervention effect hypothesised in the sample size calculation. The use of Bayes factor might, therefore, be an incentive for a more realistic and smaller estimation of anticipated intervention effects, leading to more trials with sufficient power and less trials either overestimating or underestimating intervention effects. However, if trial results confirm unrealistically large anticipated intervention effects by ‘play of chance’ there is evidently a great risk of misleading trial results. Intervention effects hypothesised in sample size calculations should therefore preferably be based on results from systematic reviews of randomised clinical trials with low risk of bias, which to some extent will ensure that realistic hypothesised intervention effects are used in the sample size calculation. If the intervention effect hypothesised in the sample size calculation is not based on results from systematic reviews of randomised clinical trials, then we recommend to calculate an additional Bayes factor using a smaller (‘sceptical’) hypothesised intervention effect, e.g., a relative risk halfway between the intervention effect hypothesised in the sample size calculation and 1.0.

Adaptive trial design has been proposed to account for the uncertainty of estimating a sample size [28]. An adaptive trial design enables sample size re-estimation at interim analyses time points during the trial [29]. At these time points the sample size can either be increased or decreased. The adaptive trial design is complex and is probably less efficient compared to the sequential design including a predefined realistic sample size [29]. Furthermore, to implement an adaptive design it should be possible, practically and financially, to expand the originally estimated sample size, which is rarely occurring in trials not financed by the industry.

Assurance is another valid method that has been proposed to estimate a sample size to achieve a desired power (assurance), rather than to achieve a desired power conditional on an assumed treatment effect [30].

### Adjustment of the confidence interval and the *P*-value when a trial is stopped before reaching the planned sample size

The majority of randomised clinical trials have difficulties in obtaining the stipulated sample size [10,31,32]. A trial that is stopped prematurely with an effect that is significant (e.g., *P* < 5%) may reach this significance level because the estimated difference in effect between the compared trial interventions is larger than anticipated or because the estimated variance is lower than anticipated — or both (see Section 2 about sample size estimation) [27,29,33]. Deviations of intervention effects far from the anticipated values should *a priori* be regarded as unlikely and this is one reason for using a lower statistical threshold to stop a trial before the planned sample size has been reached [33]. If, e.g., a sample size calculation has shown that a total of 500 patients are needed in a trial and the trial is stopped after only 250 participants are included, it might be necessary to use 1‰ instead of 5% as statistical threshold for significance in order to avoid undue declarations of statistical significance due to early random high intervention effects or low variance [34]. As mentioned, trials with too small sample sizes often show intervention effect sizes far from the effect sizes shown in larger trials and systematic reviews with meta-analyses [27,35]. As pointed out by Lindley, the apparent paradox of small trials seemingly contributing with evidence of large intervention effects while large trials tend to rule out smaller intervention effects and thereby also larger intervention effects, is bound to confuse the average clinical researcher and reader [36]. If trialists are allowed to assess statistical significance continuously during a trial (i.e., to conduct interim analyses) and stop at different time points without adjusting the level of statistical significance, this will inevitably increase the risk of falsely negating the null hypothesis [37]. This is due to sparse data and due to repetitive testing on accumulating data both leading to increased risks of random errors. Therefore, the threshold of statistical significance should be related to the fraction of the pre-planned number of participants randomised and the number of tests conducted (see also Problems with multiplicity due to multiple outcome comparisons) [38-40] — and a number of different methods have been developed for this purpose [41-44]. One example is the [41,42] O’Brien-Fleming boundaries (and the corresponding adjusted thresholds of the confidence intervals and the *P*-values), which show the adjusted thresholds for significance if a sample size has not been reached [41,45].

Any outcome should only be assessed using the thresholds used in the sample size calculation if there are sufficient data, i.e., that a sample size based on proper acceptable risks of type I and type II errors has been reached. It is, therefore, necessary to perform power calculations for all secondary outcomes (based on an anticipated intervention effect, a variance, and a risk of type I error) before randomisation begins. If an analysis of a secondary outcome has a power of less than 80%, then either the secondary outcome should be classified as an exploratory outcome or the confidence interval and the *P*-value thresholds for significance should be adjusted just as the thresholds are adjusted if a sample size has not been reached.

In conclusion, it is imperative to estimate a sufficient sample size before a trial is conducted, and proper adjustments of the thresholds for significance should be performed if a trial is stopped early or if interim analyses are conducted [17,34].

### Problems with multiplicity due to multiple outcome comparisons

If a randomised clinical trial assesses more than one outcome, compares more than two intervention groups, or assesses an outcome at more than one time point, then the overall risk of falsely rejecting the null hypothesis for at least one of the outcomes (e.g., family wise error less than 5%) may increase with the number of outcome comparisons [39]. Problems with multiplicity has major implications for the interpretation of the confidence interval and the *P*-value and this is one reason why it should be mandatory to report a predefined outcome hierarchy including a clear definition of a primary outcome before conducting a randomised clinical trial [17,40,46]. The conclusion about trial intervention effects should always be related to the result on the primary outcome (or outcomes) limiting the risk of falsely declaring a trial intervention for being effective. The outcome hierarchy and a specific, detailed description of every other relevant aspect of the trial methodology should be described in a protocol, which should be registered (e.g., at http://www.clinicaltrials.gov) and published in a journal preferably before randomisation begins [17,40,46].

How adjustment for multiplicity is done should depend on the design of the trial, i.e., the chosen outcomes and their relative importance, etc. — and different statistical methods have been proposed to adjust the observed confidence intervals and *P*-values to obtain strong control [47,48] of this risk of type 1 error when multiple outcome comparisons are used. Under weak control the type 1 error rate is controlled only under the global null hypothesis that all null hypotheses are true. Under strong control, which should be required in a clinical trial, the type 1 error rate is controlled under any partial configuration of true and false null hypotheses [47,48]. Most methods (see paragraph below) have focused on threshold adjustments and adjustments of the *P*-value, but adjusted confidence intervals can often be calculated based on an adjusted *P*-value and an effect estimate, as well as adjusted P-values can often be calculated based on adjusted confidence intervals and an effect estimate [14,49].

Adjustments of the *P*-value due to multiplicity can be obtained using Bonferroni adjustment. This simple method multiplies the *P*-value with the number of outcome comparisons when only one out of the chosen outcome comparisons must be significant in order to reject the overall null hypothesis, i.e., to declare that the trial intervention is effective [50]. The Bonferroni procedure tends to be rather conservative if the number of tests is large or if the outcomes are positively correlated. As most outcomes are dependent (e.g., incidence of cancer mortality and mortality in the same sample of participants are evidently positively correlated outcomes) Bonferroni adjustment is obviously too conservative a method to account for multiple testing and corresponding methods that are more powerful are available [51]. Hommel’s method deals with all of the chosen outcomes as a group using a data-driven adjustment of the *P*-values [52]. An alternative method (the fixed sequence procedure) is to specify the sequence of the hypothesis testing (primary outcome, first secondary, second secondary, etc.) [53]. Then each test will be done at the chosen level of significance in the specified order (here both the confidence interval and the P-value can be used to demonstrate the threshold), but as soon as a test is non-significant then the remaining null hypotheses are accepted. A fourth approach is the so-called ‘fall back procedure’ where the fixed hypothesis testing sequence is also used [54]. However, if a test is insignificant using the ‘fall back procedure’ then the procedure does not stop but the next hypothesis is tested at a reduced threshold for significance. This procedure also allows one to weight the hypotheses according to their importance and likelihood of being rejected. Other more complex methods taking correlation of the *P*-values into account are also available [55,56]. It might not be necessary to include *P*-value adjustments for outcomes pre-specified as exploratory or hypothesis generating — but such *P*-values must always be interpreted conservatively.

Analysing results from interim analyses, it may still be unknown how stable a low Bayes factor is, i.e., how often a Bayes factor once it is low will increase after additional patients have been randomised and change from below to above a certain threshold (e.g., 0.1). Full Bayesian statistics may be able to account for problems of multiplicity due to interim analyses, multiple outcomes, or comparisons of the same outcome at multiple times [57-59]. However, this may imply integration of fairly complicated models and software in the final analyses of the trial results [57-59].

### Statistical significance and clinical significance

When surrogate outcomes or continuous outcomes are used to assess intervention effects, it is often unclear if a given statistical significant effect has any patient relevant clinical significance. Moreover, if a large number of trial participants are assessed, small and clinically irrelevant intervention effects may achieve statistical significance leading to rejection of the null hypothesis [60,61]. Statins have, e.g., been widely accepted as evidence-based treatment for high cholesterol levels in the blood [62], but it has recently been shown that decades of intake of statins may only prolong life with an average of a few months [63]. For clinically relevant outcomes such as mortality, it is difficult to delineate a ‘minimal relevant clinical difference’ (Section 2). Any prevention, whatever small, of patient-important outcomes may seem relevant. Nevertheless, the significance of the clinical benefit of statins may be questioned taking adverse effects and other costs of the statins into consideration [63,64].

In spite of a statistical significant effect with even very low *P*-values and corresponding narrow confidence intervals, clinical significance can often be questioned. Relating trial results to the ‘minimal relevant clinical difference’ used to calculate the predefined sample size as well as calculating Bayes factor based on this ‘minimal relevant clinical difference’, provide indications about the clinical significance of intervention effects (see Sample size estimation, the alternative hypothesis to the null hypothesis, and Bayes factor). However, to assess the clinical significance of intervention effects it is important to perform a thorough overall assessment of the balance between beneficial and harmful effects [65,66]. Even rare serious adverse effects may rule out the rational use of an otherwise beneficial intervention [67].

It has been suggested that the ‘minimal relevant clinical difference’ should be defined as what patients perceive as important [69]. However, patients tend to regard even the smallest effect sizes as clinically important [70]. We therefore suggest that clinical researchers in close cooperation with patients and relatives must somehow consent on the quantification of the ‘minimal relevant clinical differences’ as well as the relevant outcomes to be assessed. The latter work is dealt with by research groups within The Cochrane Collaboration, The James Lind Alliance, and the COMET Initiative [14,68,71-73].

Ideally the ‘threshold’ effect size delimiting clinical significance from lack of clinical significance should, as the rest of the trial methodology, be predefined [68]. To avoid erroneous interpretations, assessment of clinical significance should only be assessed if statistical significance and a Bayes factor of less than 0.1 have been obtained.

## Discussion

The five-step procedure described aims to improve the validity of results from randomised clinical trials. The five-step procedure has the strength that is based on well-established methodology and provides a ratio of the probability that a trial result is compatible with the null hypothesis divided by the probability that the result is compatible with the intervention effect hypothesised in the sample size calculation. Our procedure adjusts for problems with multiplicity and also forces investigators and consumers of clinical research to judge clinical significance. A potential drawback of Bayesian statistical analyses is that it can be difficult to verify modelling assumptions, e.g., if assumed distributions in the analysis are appropriate or not [74]. A strength of our simplified approach is that if the assumptions behind the initial analysis methods (e.g., logistic regression or survival analysis) are fulfilled then our five-point assessment can be used validly without further testing.

The five-step procedure has limitations. First, we have provided our recommendations for understanding the results of a single randomised clinical trial in the light of usually sparse prior evidence. It has been shown that it is often unwise to base diagnostic, prognostic, preventive, or therapeutic interventions on data from one or few trials [1,9,10,26,75], and our recommendations do not in any way change this. Our aim is to introduce a simple assessment procedure which we believe will improve the validity of the assessment of results from a single randomised clinical trial, but our procedure does not solve all problems. Clinical decision-making should primarily be based on systematic reviews of all randomised clinical trials with low risk of bias including meta-analyses, trial sequential analyses, and obtained consensus of clinical significance [9,45,68,76-78]. Also in a scenario of a systematic review, calculation of Bayes factor and assessment of clinical significance may become pivotal. We will address these issues in a forthcoming article.

Second, our recommended methodology as well as our definition of Bayes factor is simplified. Alternatively, Bayes factors could be based on the ratio of the probability that the trial result is compatible with the null hypothesis divided by the probability that the result is compatible with a range of realistic alternative hypotheses. A full Bayesian analysis could also be used to analyse trial results, which focuses on the calculation of the posterior odds that an alternative hypothesis to the null hypothesis is true, given the observed data and any available prior information [2,74,79]. There are a number of major methodological advantages using full Bayesian statistics compared to frequentistic statistics [19,80] and results from a full Bayesian analysis might in some circumstances reliably show a low posterior probability for the alternative hypothesis while a low Bayes factor wrongly indicates the opposite. However, Bayesian statistical analyses increases the methodological complexity [19,80]; can make research results sensitive to apparently innocuous assumptions which hinder taking possible trial results into account [80,81]; and will, in essence, require a methodological paradigm shift including use of detailed Bayesian statistical analyses plans and Bayesian statistical software such as WinBUGS [82].

Third, it is necessary to define some kind of alternative hypothesis to the null hypothesis calculating the Bayes factor. The definition of the alternative hypothesis often involves an element of subjectivity, and it is for this reason that many trialists do not use the Bayesian approach [2,79]. It has been suggested that the alternative hypothesis might be defined as ‘uniformly most powerful Bayesian tests’ where the alternative hypothesis is defined as an average value of any hypothetical intervention effect resulting in a Bayes factor below a given threshold [2,79]. This procedure is appealing because no subjective assumptions have to be made about the alternative hypothesis — but it is a problem that potentially important information about intervention effects showed in former randomised trials or systematic reviews of such trials cannot be included in the definition of the alternative hypothesis. Furthermore, the method is primarily for one-parameter exponential family models and has, in essence, no methodological advantages compared to only using the *P*-value as a threshold for significance [2,79]. The researcher behind the ‘uniformly most powerful Bayesian tests’ suggests to use lower *P*-value thresholds (0.005 or 0.001) to avoid false positive significant results [2], which clearly seems to be a valid alternative to our calculation and use of Bayes factor. We have chosen the intervention effect hypothesised in the sample size calculation as the alternative hypothesis firmly relating the pre-planned trial design to the interpretation of the trial result. Most trials already include a predetermined sample size calculation, which includes estimation of an anticipated intervention effect. New assumptions are therefore, in essence, not needed to calculate Bayes factor. However, it is still a clear limitation that Bayes factor can be influenced by *post-hoc* adjustments and erroneous quantifications of the alternative hypothesis.

Fourth, our procedure is based on already well-established methodology. However, there is no empirical evidence so far assessing the validity of the procedure. We will also address this issue in a forthcoming article.

## Conclusions

To assess the statistical significance and the clinical significance of results from randomised clinical superiority trials, we propose a five-step procedure: (1) Calculate and report the confidence intervals and the exact *P*-values for all pre-specified outcome comparisons. A *P*-value less than 0.05 may be chosen as threshold for statistical significance for the primary outcome, only if 0.05 has been used as the acceptable risk of type I error in the sample size calculation and the sample size has been reached. (2) Calculate and report the Bayes factor for the primary outcome (or outcomes) based on the hypothesised intervention effect used in the sample size estimation. If the intervention effect hypothesised in the sample size calculation is not based on results from systematic reviews or randomised clinical trials, then calculate an additional sceptical Bayes factor using a smaller hypothesised intervention effect, e.g., a relative risk halfway between 1.0 and the intervention effect hypothesized in the sample size calculation. A Bayes factor less than 0.1, indicating a ten-fold higher likelihood of compatibility with the alternative hypothesis than the likelihood of compatibility with the null hypothesis, may be chosen as threshold for supporting the alternative hypothesis. More research is needed to assess if this threshold is optimal. (3) If the *a priori* estimated sample size has not been reached or interim analyses have been performed, then adjust the confidence intervals and the *P*-values accordingly. (4) If more than one outcome is used, if more than two intervention groups are compared, or if the primary outcome is assessed at multiple time points (and just one of these outcome comparisons must be significant to reject the overall null hypothesis), then the confidence intervals and the *P-*values should be adjusted accordingly. (5) Assess and report clinical significance of the results if all of the first four steps of the five-point procedure have shown statistical significance.

Table 1 summarises our suggestions for a more valid assessment of intervention effects in randomised clinical superiority trials, and we have included three examples of how the five-step assessment can be used to assess statistical significance and clinical significance of results from a randomised clinical trial (see Example 1, Example 2, Example 3). We have, for simplicity, only assessed the primary outcome results in the three examples.

**Table 1:** Our suggestions for a more valid assessment of intervention effects in a randomised clinical superiority trial

1 Calculate and report the confidence intervals and the exact *P*-values for each pre-specified outcome.	
2 Calculate and report the Bayes factor (see Additional file 1: Table S1 for calculations) for the primary outcome. A Bayes factor less than 0.1 may be chosen as threshold for significance.	
3 If the a priori estimated sample size has not been reached or if interim analyses have been conducted, then adjust the confidence intervals and the *P*-values accordingly.	
4 If more than one outcome is used, if more than two intervention groups are compared, or if the primary outcome is assessed multiple times (and just one of these outcome comparisons must be significant to reject the overall null hypothesis), then the confidence intervals and the *P-*values should be adjusted accordingly.	
5 If statistical significance has been obtained according to all of the first four points above then assess clinical significance of the trial results.	

A low Bayes factor (e.g., less than 0.1) together with a low *P*-value (e.g., less than 0.05) will correspond to a high probability of an intervention effect similar to or greater than the hypothesised intervention effect used in the sample size calculation.

All of these aspects should be prospectively planned and published in a public protocol for the randomised clinical trial before inclusion of the first participant.

### Example 1

A trial published in JAMA 2012 examined the effects of multivitamins in the prevention of cancer [83]. The conclusion of the trial was that multivitamin supplementation significantly reduced the risk of total cancer (HR 0.92; 95% CI, 0.86 to 0.998; P = 0.04). We will use our five-step procedure to assess the statistical and clinical significance of the trial results:

1. *Report the confidence interval and the exact P-value.*

Our assessment: The hazard ratio, the 95% confidence interval, and the exact *P*-value are reported in the publication (HR 0.92; 95% CI, 0.86 to 0.998; P = 0.04).

2. *Calculate and report the Bayes factor for the primary outcome. A Bayes factor less than 0.1 may be chosen as threshold for significance.*

Our assessment: First, to calculate Bayes factor we need to calculate log odds ratio and the standard error of the log odds ratio of the trial result: odds ratio 0.92, log odds ratio −0.08, and standard error of the log odds ratio 0.04.

Second, we need to calculate the log odds ratio of the sample size calculation. The statistical power for showing a 20% and a 30% reduction in the risk of total cancer was calculated in the protocol [84]: odds ratio 0.8 and log odds ratio −0.22.

Bayes factor = 53.10 if a risk reduction of 20% is used as the anticipated intervention effect, which is considerably greater than 0.1.

Bayes factor = 3,009,380,258 if a risk reduction of 30% is used as the anticipated intervention effect, again considerably greater than 0.1.

3. *If the a priori estimated sample size has not been reached or if interim analyses have been performed, then adjust the confidence intervals and the P-values accordingly.*

Our assessment: The sample size estimation is based on a total of 15,000 participants, and 14,641 participants are randomised in the trial. The sample size was almost sufficiently reached, so no adjustment may be needed.

4. *If more than one outcome is used, if more than two intervention groups are compared, or if the primary outcome is assessed multiple times (and just one of these outcome comparisons must be significant to reject the overall null hypothesis), then the confidence intervals and the P-values should be adjusted accordingly*.

Our assessment: In the published protocol [84] it is reported that the trial is a randomised, double-blind, placebo-controlled trial of the balance of benefits and risks of beta-carotene, vitamin E, vitamin C, and a multivitamin in the prevention of total and prostate cancer, cardiovascular disease, and the age-related eye diseases, cataract and macular degeneration. In the protocol no clear definition of a primary outcome is reported [84]. Five outcomes (total cancer, prostate cancer, important cardiovascular events, age-related macular degeneration, and cataract) are mentioned in the protocol to assess all the included outcomes. However, in the trial publication total cancer is mentioned as the primary outcome. The *P*-value of 0.04 should properly have been adjusted for multiplicity due to the many outcome comparisons.

5. *If statistical significance has been shown according to all of the above points then assess clinical significance of the trial results.*

Our assessment: The assessment of statistical significance was not adequately addressed and if it had been it is highly unlikely if statistical significance would have been attained. So it is not deemed relevant to assess any clinical significance.

Interpretation: Our five-point assessment demonstrates that the results from the randomised clinical trial should be interpreted with great caution and that the results from this single trial indicates that the effect of multivitamins is 53 times more compatible with the null hypothesis than the hypothesis of a 20% relative risk reduction of total cancer. Our five-point assessment of this trial is in agreement with results from systematic reviews with meta-analysis and trial sequential analysis on all-cause mortality, gastrointestinal cancers, and other cancers [85-87].

### Example 2

A trial published in The Lancet 2010 examined the effects of tranexamic acid versus placebo in trauma patients with significant haemorrhage [88]. The conclusion of the trial was that tranexamic acid significantly reduced all-cause mortality. We will use our five-step procedure to assess the statistical and clinical significance of the trial results:

1. *Report the confidence interval and the exact P-value.*

Our assessment: The authors reported a relative risk 0.91, 95% CI 0.85 to 0.97, and *P* = 0.0035

2. *Calculate and report the Bayes factor for the primary outcome. A Bayes factor less than 0.1 may be chosen as threshold for significance.*

Our assessment: First, to calculate Bayes factor we need to calculate the log odds ratio and the standard error of the log odds ratio of the trial result: odds ratio 0.89, log odds ratio −0.12, and standard error of the log odds ratio 0.04.

Second, we need to calculate the log odds ratio of the intervention effect hypothesised in the sample size calculation. The sample size calculation was based on an assumed risk of death of 20% in the control group, a relative risk of 0.90, and it was planned to randomise 2 × 10,000 participants. This corresponds to an odds ratio of 0.89 and a log odds ratio of −0.11.

$$\mathrm{Bayes}\;\mathrm{factor}=\begin{array}{c} \exp\left[-\begin{array}{c} 1\\ 2\times 0.04^{2} \end{array}{\left(-0.12\right)}^{2}\right]\\ \exp\left[-\begin{array}{c} 1\\ 2\times 0.04^{2} \end{array}{\left(-0.12-\left(-0.11\right)\right)}^{2}\right] \end{array}$$

Bayes factor = 0.01 which is 10 times less than the suggested threshold of 0.1. Accordingly, there seems to be good support of the assessed postulated intervention effect.

3. *If the a priori estimated sample size has not been reached or if interim analyses have been performed, then adjust the confidence intervals and the exact P-values accordingly*

Our assessment: The sample size estimation is based on a total of 20,000 participants, and 20,211 were randomised in the trial. The sample size was sufficiently reached.

4. *If more than one outcome is used, if more than two intervention groups are compared, or if the primary outcome is assessed multiple times (and just one of these outcome comparisons must be significant to reject the overall null hypothesis), then the confidence intervals and the P-values should be adjusted accordingly.*

Our assessment: Only one primary outcome is reported in the published protocol (http://www.thelancet.com/protocol-reviews/05PRT-1) and this outcome is only assessed at one time point. There is no need for an adjustment of the confidence interval or the *P-*value.

5. *If statistical significance has been shown according to all of the above four points, then assess clinical significance of the trial results.*

Our assessment: Statistical significance was reached according to all of the first four points of the five-point assessment. Clinical significance for a dichotomous outcome can be assessed by calculating the number-needed-to-treat to save one life. Number-needed-to-treat is 35 participants, demonstrating a relatively large clinical benefit of tranexamic acid. A further assessment of the balance between beneficial and harmful effects should also be performed.

Interpretation: Our five-point assessment demonstrates that the results from the randomised clinical trial are a 100 times more compatible with a 10% relative risk reduction than the null effect of tranexamic acid on all-cause mortality. However, before this promising treatment is introduced into clinical practice, a systematic review of all randomised clinical trials should assess the benefits and harms of tranexamic acid. Such a review should include a thorough bias risk assessment, meta-analyses, trial sequential analyses, and reports on harm from observational studies [9,45,68,76-78].

### Example 3

A trial published in The New England Journal of Medicine in 2012 examined the effects of hydroxyethyl starch versus Ringer’s acetate in severe sepsis [89]. The conclusion of the trial was that the primary outcome, death or dependence on dialysis at 90 days after randomisation, occurred in 202 patients (51%) in the starch group as compared with 173 patients (43%) in the Ringer’s acetate group (relative risk, 1.17; 95% CI, 1.01 to 1.36; P = 0.03).

We will use our five-step procedure to assess the statistical and clinical significant of the trial results:

1. *Report the confidence interval and the exact P-value.*

Our assessment: The confidence interval and the *P-*value are reported in the publication (relative risk, 1.17; 95% CI, 1.01 to 1.36; P = 0.03).

2. *Calculate and report the Bayes factor for the primary outcome. A Bayes factor less than 0.1 may be chosen as threshold for significance.*

Our assessment: First, to calculate Bayes factor we need to calculate the log odds ratio and the standard error of the log odds ratio of the trial result: odds ratio 1.35, log odds ratio 0.30, and standard error of the log odds ratio 0.142.

Second, we need to calculate the log odds ratio of the sample size calculation. The sample size calculation reported in the published protocol showed that a total of 800 participants was needed to show a 20% relative risk reduction on either death or end-stage kidney failure (primary outcome) assuming a 50% incidence of either death or end-stage kidney failure in the control group. This will correspond to an odds ratio of 0.67 and a log odds ratio of −0.40.

Bayes factor = 20,306 which is considerably greater than 0.1.

It must be noted that the trialists anticipated a beneficial effect of hydroxyethyl starch, but found a harmful effect compared with Ringer’s acetate. This results in a large Bayes factor demonstrating that the trial results show that it is far more probable that the results are compatible with a null effect (or a harmful effect) than the results are compatible with a 20% relative risk reduction of mortality hypothesised in the sample size calculation.

3. *If the a priori estimated sample size has not been reached or if interim analyses have been performed, then adjust the confidence intervals and the exact P-values accordingly.*

Our assessment: The sample size estimation is based on a total of 800 participants, and 804 participants were randomised. The sample size was reached.

4. *If more than one outcome is used, if more than two intervention groups are compared, or if the primary outcome is assessed multiple times (and just one of these outcome comparisons must be significant to reject the overall null hypothesis), then the confidence intervals and the P-values should be adjusted accordingly.*

Our assessment: The same single primary outcome (either death or end-stage kidney failure) was reported in the published protocol [90] and in the trial publication [89]. The primary outcome was only planned to be analysed at one time point. There is no need for any adjustment of the threshold for significance.

5. *If statistical significance has been shown according to the above four points, then assess clinical significance of the trial results.*

Our assessment: The first four points of the five-point assessment clearly showed that hydroxyethyl starch does not seem to have a beneficial effect. Clinical significance can for dichotomous outcomes be assessed by calculating number-needed-to-treat or number-needed-to-harm. The number-needed-to-harm is 13, i.e., after 13 patients with severe sepsis have been treated with hydroxyethyl starch one extra patient will die or develop end-stage renal disease because of being treated with hydroxyethyl starch compared with being treated with Ringer’s acetate. A further assessment of the balance between beneficial and harmful effects should also be performed but is irrelevant in this trial.

Interpretation: Our five-point assessment confirms that hydroxyethyl starch compared with Ringer’s acetate does not seem to have a beneficial effect in the treatment of severe sepsis. Our five-point assessment is in agreement with results from systematic reviews with meta-analysis and trial sequential analysis [91].

## Competing interests

The authors declare that they have no competing interests.

## Authors’ contributions

JCJ wrote the first draft. All authors were substantially involved in revising the manuscript and all authors have given final approval of the present version to be published.

## Pre-publication history

The pre-publication history for this paper can be accessed here:

http://www.biomedcentral.com/1471-2288/14/34/prepub

## Supplementary Material

Additional file 1: Table S1 Different statistical terms and calculation of Bayes factor. Click here for file
