# Supplementary material for: The thresholds for statistical and clinical significance – a five-step procedure for evaluation of intervention effects in randomised clinical trials
Source: BMC Med Res Methodol. 2014 Mar 4;14:34. doi: 10.1186/1471-2288-14-34 (PMC4015863; doi:10.1186/1471-2288-14-34)
Supplement: Additional file 1: Table S1 — Different statistical terms and calculation of Bayes factor. [file 1471-2288-14-34-S1.docx]

**Table S1**

| **Different statistical terms and calculation of Bayes factor** | |
| --- | --- |
| **Null hypothesis** | The hypothesis that there is no difference in effect between the compared interventions. |
| ***P*** | A function whose value is the probability of obtaining a specified result or one consistent with a larger effect of the intervention, given there is no effect. |
| **Bayes factor** | A measure of the relative likelihood of two hypotheses [19].  Pr = probability density; DataObs = observed data; ⏐=given; H_0_ = null hypothesis is true; H_A_ = alternative hypothesis is true. |
| **Obtainment of the formula for Bayes factor** | For an asymptotically normally distributed effect size estimate (e.g., a mean difference, a log odds ratio, or a log hazard ratio) Bayes factor can be calculated using the following:  |
| **Calculating Bayes factor** |  or the more simple form:    = the intervention effect hypothesised in the sample size calculation (e.g., a mean difference (continuous outcome), a log odds ratio (dichotomous outcome), or a log hazard ratio);= difference in effect shown by the observed data; SE = standard error of .  The standard error of the log odds ratio can be calculated using data from a standard 2x2 table for dichotomous outcomes:   \|  \| Event of interest \| No event of interest \| \| --- \| --- \| --- \| \| Experimental group \| a_i_ \| b_i_ \| \| Control group \| c_i_ \| d_i_ \|     |
